# Altered p16^Ink4a^, IL-1β, and Lamin b1 Protein Expression Suggest Cellular Senescence in Deep Endometriotic Lesions

**DOI:** 10.3390/ijms23052476

**Published:** 2022-02-24

**Authors:** Helena Malvezzi, Cristine Dobo, Renee Zon Filippi, Helen Mendes do Nascimento, Laura Palmieri da Silva e Sousa, Juliana Meola, Carla Azevedo Piccinato, Sérgio Podgaec

**Affiliations:** 1 Hospital Israelita Albert Einstein, Av. Albert Einstein 627, Morumbi 05652-900, SP, Brazil; cristine.dobo@einstein.br (C.D.); renee.filippi@einstein.br (R.Z.F.); helen2002@me.com (H.M.d.N.); laurapalmieri1998@gmail.com (L.P.d.S.e.S.); cpiccinato@uwalumni.com (C.A.P.); sergiopodgaec@me.com (S.P.); 2 School of Medicine of Ribeirão Preto, University of São Paulo, Gynecology and Obstetrics, Av. Bandeirantes, 3900, Vila Monte Alegre 14049-900, SP, Brazil; jumeola@yahoo.com.br

**Keywords:** senescence, endometrium, deep endometriotic lesion, senescence-associated secretory phenotype, inflammatory cytokines

## Abstract

Endometriosis causes immunological and cellular alterations. Endometriosis lesions have lower levels of lamin b1 than the endometrium. Moreover, high levels of pro-inflammatory markers are observed in the peritoneal fluid, follicular fluid, and serum in endometriosis lesions. Thus, we hypothesized that the accumulation of senescent cells in endometriosis tissues would facilitate endometriosis maintenance in an inflammatory microenvironment. To study senescent cell markers and the senescence-associated secretory phenotype (SASP) in endometriosis lesions, we conducted a cross-sectional study with 27 patients undergoing video laparoscopy for endometriosis resection and 19 patients without endometriosis. Endometriosis lesions were collected from patients with endometriosis, while eutopic endometrium was collected from patients both with and without endometriosis. Tissues were evaluated for senescence markers (p16^Ink4a^, lamin b1, and IL-1β) and interleukin concentrations. The expression of p16^Ink4a^ increased in lesions compared to that in eutopic endometrium from endometriosis patients in the secretory phase. In the proliferative phase, lesions exhibited lower lamin b1 expression but higher IL-4 expression than the eutopic endometrium. Further, IL-1β levels were higher in the lesions than in the eutopic endometrium in both the secretory and proliferative phases. We believe that our findings may provide targets for better therapeutic interventions to alleviate the symptoms of endometriosis.

## 1. Introduction

Endometriosis is a chronic inflammatory gynecological disease with no clear etiology [1]. An etiopathogenesis theory for the development of endometriosis is that endometriosis is caused by immunological alterations and changes in cellular behavior. Higher concentrations of inflammatory cells are present in the peritoneal and follicular fluid [2,3], serum [4], and menstrual reflux [5,6,7]. Further, levels of inflammatory products are higher in women with endometriosis.

Some processes that control implantation, development, and maintenance of endometrial-like cells in the peritoneal cavity are well described. For instance, apoptosis resistance, angiogenesis increase [8,9], and proliferative capacity [10] are influenced by high concentrations of pro-inflammatory cytokines and growth factors, such as transforming growth factor, interleukin (IL)-1, IL-6, IL-8, tumor necrosis factor (TNF), interferon-gamma [11,12,13], and by recruitment of immune system cells, such as macrophages [14], natural killer cells [15,16], and neutrophils [17,18]. However, it is unclear whether this inflammatory pattern is a cause or a consequence of the implantation and development of endometrial tissue outside the uterus. Further, senescent cells are capable of secreting growth factors, extracellular matrix components, and inflammatory cytokines, in addition to resisting apoptosis and exhibiting a long lifespan [19,20,21,22]. Senescence is an irreversible process of cell cycle suspension. However, senescent cells remain active indefinitely, synthesizing metabolites that characterize the senescence-associated secretory phenotype (SASP) [23,24].

Senescence-related secretory factors are associated with chronic inflammation, a feature of endometriosis. Although the SASP can help tissue regeneration and immune cell recruitment in a damaged area, an incessant SASP may be more hazardous than beneficial by promoting proliferation, angiogenesis, and inflammation [25]. Currently, several biomarkers are used to monitor senescent cells in a given tissue both in vitro and in vivo, such as p16^Ink4a^ and lamin b1 [26,27,28]. Interestingly, we observed a decrease in lamin b1 expression in endometriosis lesions compared to that in the eutopic endometrium [29]. Moreover, some cytokines known to be present in the SASP, namely IL-6, IL-8, IL-1β, and IL-15 [25,30], which significantly overlap with secreted cytokines related to chronic endometriosis [31,32].

These data led us to hypothesize that there is an accumulation of senescent cells in endometriosis tissues (lesions and endometrium) and that cellular senescence would facilitate the maintenance of endometriosis through cell cycle control, which is further favored by an inflammatory microenvironment related to the SASP. The latter would induce the establishment of more senescent cells in endometriosis lesions and therefore hinder both local apoptosis and inflammation, which are characteristics of the disease. Hence, in this study, we explored this hypothesis by evaluating a group of key senescence markers, namely p16^Ink4^, IL-1β, lamin b1, and the SASP, in tissues of women with deep endometriosis. To the best of our knowledge, the investigation of the relationship between endometriosis and senescence is novel, and only a few studies have linked the presence of senescence markers with symptoms or physiopathogenesis of the disease. Thus, we investigated the altered presence of three senescent cells markers in deep endometriosis lesions and assessed the cellular localization of two of these proteins.

## 2. Results

### 2.1. Patient Characteristics

Although only retrocervical lesions were collected for this study, the number of patients with different types of concomitant lesions were as follows: diaphragmatic (1 patient, 4%), ovarian endometriomas (12 patients, 44%), superficial (19 patients, 70%), periureteral (three patients, 11%), rectosigmoid (11 patients, 41%), and vaginal (15 patients, 56%).

Dysmenorrhea was the most common symptom among the endometriosis (24.89%) and non-endometriosis (11.56%) groups. Patients from both the endometriosis and non-endometriosis groups had other complaints, such as chronic pelvic pain (52% and 19%, respectively), deep dyspareunia (74% and 19%, respectively), intestinal symptoms (67% and 0%, respectively), urinary symptoms (11% and 0%, respectively), and infertility (0% and 6%, respectively).

In the endometriosis group, 15 patients were in the proliferative phase and 12 were in the secretory phase. The non-endometriosis group consisted of 10 patients in the proliferative phase and 9 in the secretory phase.

### 2.2. Age and Body Mass Index Do Not Correlate with Senescence Markers

Since we evaluated a cell type (senescent cell) that could change according to age and body mass index (BMI), we first compared age and BMI between all subgroups, considering the secretory and proliferative phases. The mean (CI) age (in years) and BMI (in kg/m^2^) in the secretory phase were 41.7 (38.7–44.9) and 29.0 (26.4–31.7), respectively, in the non-endometriosis group, and 35.8 (33.2–38.5) and 23.4 (21.6–25.3), respectively, in the endometriosis group. The mean (CI) age (in years) and BMI (in kg/m^2^) in the proliferative phase were 38.9 (36.1–42.0) and 24.0 (22.0–26.2), respectively, in the non-endometriosis group, and 35.5 (33.2–38.0) and 22.2 (20.7–23.9), respectively, in the endometriosis group. The results were statistically different during the secretory phase between the non-endometriosis and endometriosis groups for age and BMI (*p* = 0.023 and *p* = 0.004, respectively). However, we did not find any correlation between age or BMI and the levels of senescence markers (p16^Ink4a^, lamin b1, and IL-1β). To investigate the relationship between age and BMI with the protein expression of senescence markers in tissue samples, we adjusted the models considering the dependence between different tissues from the same patient; the results are shown in Table 1.

### 2.3. Increased Expression of Senescence Markers in Endometriosis Lesions Compared to Eutopic Endometrium

**Figure 1:**
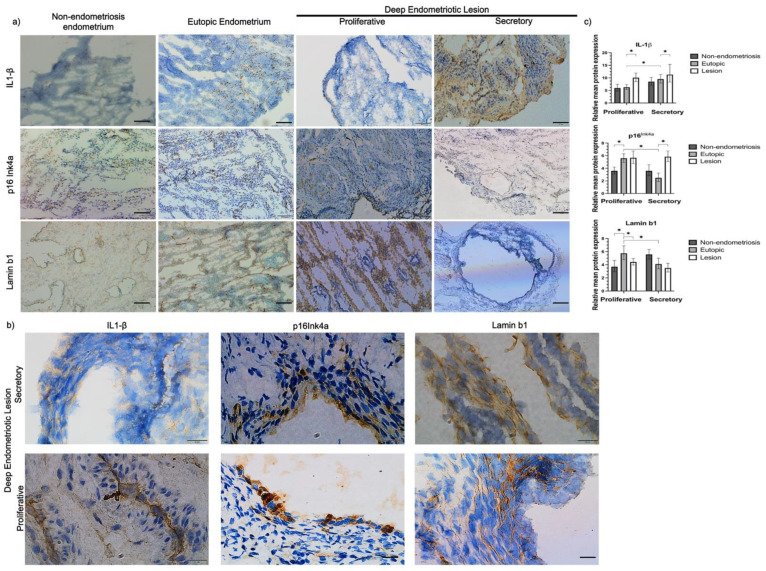
(**a**) Representative images of p16^Ink4a^, lamin b1, and IL-1β staining on endometriosis tissues and non-endometriosis. The brown color represents the areas stained by the anti-p16ink4a, anti-lamin b1, and IL-1β antibodies; the hematoxylin (blue color) marks the cell nuclei; and the white spaces represent spaces with no cells. i Ovary, positive reaction control; ii ovary, negative reaction control; iii non-endometriosis endometrium; iv eutopic endometrium; v endometriosis lesions. Pictures were acquired at 20X magnification. Scale: 100 µm. (**b**) Representative images of p16^Ink4a^ and lamin b1 staining in deep endometriosis lesion tissues. The brown color represents the areas stained by the p16^Ink4a^, lamin b1, and IL-1β antibodies; the blue color (DAPI—4′,6-diamidino-2-phenylindole) marks the cell nuclei. Images suggest the presence of stromal cells (red circle) and epithelial cells (yellow star) in deep endometriosis lesions. I Lamin b1-stained deep endometriosis lesions; ii p16^Ink4a^-stained deep endometriosis lesions; iii IL-1β-stained deep endometriosis lesions. Pictures were acquired at 20X and 100X magnifications. Scale: 10 µm. (**c**) Protein expression of p16^Ink4a^, lamin b1, and IL-1β, compared between control, eutopic endometrium, and lesion. The percentage represents the total area marked by the tissue in each image. The average of each of the 16 images for each tissue was calculated, and the value of this average was used to calculate the difference between the groups. * *p* < 0.05. Results are presented as mean and confidence interval estimated from generalized linear models of the gamma family.

Representative immunohistochemistry images of all tissues analyzed for p16^Ink4a^, lamin b1, and IL-1β expression are shown in Figure 1a and b. In lesions, we observed 1.57-fold and 1.6-fold increases in p16^Ink4a^ and IL-1β protein expression, respectively, compared to that in the eutopic group in the secretory phase. However, no difference in lamin b1 expression between the groups during the secretory phase (Figure 1c) was detected. In the proliferative phase, IL-1β expression in the lesion group was increased 1.72-fold in relation to the eutopic group, and the lamin b1 expression in the lesion group was 36% lower than that in the eutopic endometrium group; however, we did not detect a significant difference in p16^Ink4a^ expression among the groups (Figure 1c). The detailed results are shown in Table 2.

### 2.4. p16^Ink4a^ and Lamin b1 Are Expressed in Both Glandular and Non-Glandular Epithelial Cells

**Figure 2:**
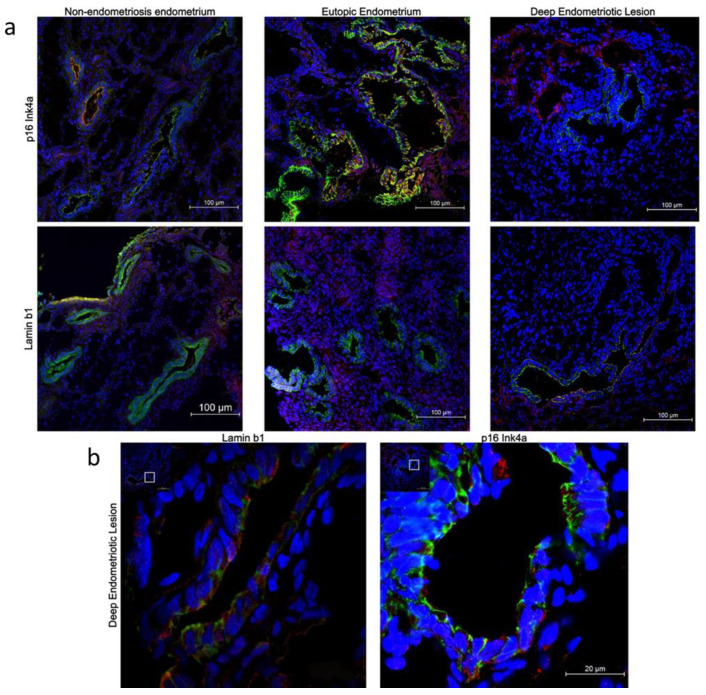
(**a**) Representative images of endometriosis and non-endometriosis tissues co-stained with p16^Ink4a^ or lamin b1 and e-cadherin (epithelial cell marker). p16^Ink4a^ and anti-lamin b1 are shown in red, e-cadherin in green, and the blue color (DAPI—4′,6-diamidino-2-phenylindole) marks the cell nuclei. (i) non-endometriosis endometrium; (ii) eutopic endometrium; (iii) endometriosis lesions. Pictures were acquired at 20× magnification. (**b**) Representative images of p16^Ink4a^ and (**c**) lamin b1 co-stained with e-cadherin in deep endometriosis lesion tissues. p16^Ink4a^ and anti-lamin b1 are shown in red, e-cadherin in green, and the blue color (DAPI—4′,6-diamidino-2-phenylindole) marks the cell nuclei. The red arrow shows cells that are only stained for p16^Ink4a^ or lamin b1, and the yellow star shows epithelial cells stained for both p16^Ink4a^ and lamin b1. Pictures were acquired at 20× and 100× magnifications.

Tissues from non-endometriosis control and endometriosis patients had similar senescence marker staining results (Figure 2a,b). A portion of p16^Ink4a^- and lamin b1-positive cells were glandular epithelial cells; however, senescence-positive cells could also be observed in non-glandular epithelial cells (Figure 2b).

### 2.5. Inflammatory Cytokine Expression in Endometriosis Lesions Was Lower Than That in Eutopic Endometrium in the Secretory and Proliferative Phases

**Figure 3:**
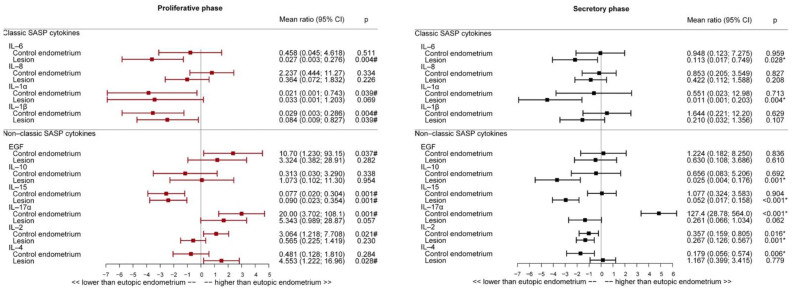
Senescence-associated secretory phenotype (SASP) and cytokine expression (pg/mL) according to tissue type (non-endometriosis endometrium, eutopic endometrium, and endometriosis lesion). * *p* < 0.05, compared to the eutopic endometrial group in the secretory phase. # *p* < 0.05, compared to the eutopic endometrial group in the proliferative phase.

Expression of cytokines IL-2, IL-6, IL-10, and IL-15 was lower in endometriosis lesions than in eutopic endometrium in the secretory phase (Figure 3). IL-1β, IL-6, and IL-15 expressions were lower, and IL-4 was higher in endometriosis lesions compared to that in eutopic endometrium in the proliferative phase (Figure 3). However, no difference was detected in EGF, IL-1α, IL-2, IL-17A, and IL-8 expression between the groups in either menstrual phase.

### 2.6. Interleukin Expression Profile in Eutopic and Non-Endometriosis Endometrium

The comparison between eutopic and non-endometriosis endometrium revealed that in the proliferative phase (Figure 3), the expression of IL-15 in non-endometriosis endometrium decreased by 92.3% in relation to that in the eutopic endometrium, whereas IL-1α and IL-1β expression decreased by 97.9% and 97.1%, respectively. The expression of IL-2, IL-17A, and EGF was higher in the non-endometriosis group than in the eutopic endometrium group. IL-4 levels were not significantly different between the non-endometriosis and eutopic groups in the proliferative phase.

In the secretory phase (Figure 3), IL-2 and IL-4 expression decreased by 64.3% and 82.1%, respectively, in the non-endometriosis endometrium, compared to the eutopic endometrium. IL-17A levels increased by more than 100 times in the control group. No difference in EGF, IL-1α and β, and IL-15 expression was observed between the control and eutopic endometrium groups. Further, we found no difference in IL-6, IL-8, and IL-10 expression between the eutopic and non-endometriosis endometrium in either menstrual cycle phase.

### 2.7. Correlation between Cytokines and Senescence Markers in Eutopic Endometrium and Endometriosis Lesions in the Proliferative and Secretory Phases

When evaluating the correlation between senescence markers and cytokine expression (Table 3), we found that in the secretory phase, with an increase of one unit in the expression of cytokine IL-17A, the p16^Ink4a^ mean is expected to increase by 3.6%. With the increase of one unit in the expression of cytokines IL-1α and IL-2, the lamin b1 mean is expected to decrease by 0.1% and 70%, respectively.

## 3. Discussion

In this study, we investigated a plethora of cellular senescence markers and the SASP in deep endometriosis lesions and eutopic endometrium. Cellular senescence is characterized by a permanent state of cell cycle arrest in response to various cell-damaging stimuli [21]. If not eliminated, senescent cells accumulate in a given organ or tissue and may favor a pro-inflammatory environment, thereby decreasing the regenerative capacity of the cell and favoring the appearance of several diseases, such as cancer, degenerative diseases, and aging syndromes; this is known as chronic senescence [33].

The main characteristics of cellular senescence include the release of pro-inflammatory cytokines, the expression of anti-apoptotic genes and oncogenes, and changes in metabolic rates [34]. However, in the absence of a universal hallmark [22,33], cellular senescence can be assessed through the analysis of senescence markers, such as p16^Ink4a^ [35], lamin b1 [36], and IL-1β [37]. The present study demonstrated that in the same patient, the retrocervical deep-endometriosis lesion has pro-senescence characteristics, as shown by the presence of higher IL-1β and p16Ink4a expression and lower lamin b1 expression, compared to the eutopic endometrium.

IL-1α and IL-1β are known components of the SASP [38] and are the main precursor proteins that bind IL-1R1. Activated IL-1α and 1β initiate a signaling cascade that leads to nuclear translocation of the transcription factor NF-κB, resulting in numerous immune effects, such as the production and secretion of cytokines, chemokines, and growth factors; upregulation of adhesion molecules; increase in vascular permeability; and proliferation of effector T cells in the presence of regulatory T cells [37,39]. We demonstrated that endometriosis lesions exhibited higher expression of IL-1β than eutopic endometrium, corroborating our hypothesis of upregulated senescence marker expression in endometriosis lesions. Although IL-1 is a minor SASP component, it is responsible for initiating a cascade of events that ultimately leads to IL-6 and IL-8 secretion, both of which are major components of the SASP [40]. In this study, although we could not find any difference in IL-6 and IL-8 levels between endometriosis lesions and eutopic endometrium, we noticed a disruption in IL-1α and IL-1 production, suggestive of endometriosis cells entering the senescence state.

The p16^Ink4a^ protein is critical for regulating aging and cell senescence, detecting and maintaining DNA damage, and inhibiting cell proliferation [41]. Studies in murine models suggest that p16^Ink4a^ is associated with the loss of cellular replication and accumulation of senescent cells [42,43]. This leads to a permanent cell cycle arrest, mainly through the persistent inhibition of Cdk–cyclin activity by p16^Ink4a^ [44]. Similar to our results, increased p16^Ink4a^ expression was found in ovarian and endometrial cancer as well as in endometriotic ovarian cysts [45,46,47]. The significantly higher p16^Ink4a^ expression could be explained by oncogene-induced cellular senescence. Since there are conflicting results in the literature, and the association between p16^Ink4a^, endometriosis, and senescence has only relatively recently been identified, this topic needs further investigation [48]. Recently, the decrease in p16^Ink4a^-positive cells was correlated with higher implantation failure and miscarriage among women submitted to in vitro fertilization (IVF) procedures [49]. This result could be due to naturally occurring reduced telomerase activity (TA) during the secretory phase of the menstrual cycle [50], since p16 transfection suppressed TA [51]. Thus, increased p16 expression encountered in IVF pregnant patients from the Parvanov et al. (2021) study could be suppressing TA and facilitating embryo implantation. In contrast, in eutopic endometrium from endometriosis patients, there is an increase in telomerase activity during the secretory phase of the menstrual cycle [50], which may explain the high survival and proliferative capacity of ectopic cells in the peritoneal cavity.

In our previous study, we demonstrated a decrease in lamin b1 expression in endometriosis lesions compared to that in the eutopic endometrium; however, we did not consider the different phases of the menstrual cycle [29]. In this study, in an enlarged subset of patients, we found a difference in lamin b1 expression during the proliferative phase, where lamin b1 concentration was lower in endometriosis lesions than in the eutopic endometrium. Lamin b1 is associated with the maintenance of the nuclear membrane structure, DNA replication, and chromatin organization [52]. Moreover, lamin b1 modifies the genes responsible for cell-cycle control, a phenomenon that corroborates the senescence-associated growth arrest [53], while other studies have also associated low lamin b1 levels with cellular senescence [52,54,55]. A recent study on induced senescence in HeLa cells demonstrated that lamin b1 knockdown resulted in the upregulation of SASP factors such as IL-6, IL-8, and metalloproteinase 1, suggesting that lamin b1 might also be crucial in SASP regulation [56].

Our results revealed a positive correlation between IL-17A and p16^Ink4a^ expression and a negative correlation between lamin b1 expression and that of IL-1α and IL-2 in the secretory phase. These traits might help in maintaining a pro-inflammatory environment that would favor a pro-senescent milieu. However, the direct effect of IL-17A on p16^Ink4^ induction requires further investigation.

As stated earlier, cellular senescence is a dynamic process [57] that leads to permanent cell cycle arrest [44]. Further, the downregulation of lamin b1 triggers modifications in chromatin methylation, generating profound transcriptional changes. This might modify the expression of genes responsible for cell cycle control, which might lead to a senescence-associated growth arrest [53]. Furthermore, there is an upregulation of IL-1β, a cytokine associated with the start of senescence. In lesions, we observed higher expression of p16^Ink4a^ and IL-1β and lower expression of lamin b1 than in the eutopic endometrium, which indicates that senescent cell biomarkers are present in endometriosis lesions. We believe that the presence of senescence markers in such lesions might be correlated with cellular behavior modifications, which in turn are associated with senescent cell accumulation. This observation is also consistent with the pro-inflammatory milieu characteristic of the disease.

Senescence is a permanent, irreversible cellular state [44]; thus, differences found in senescence markers, including the SASP, might be due to epithelial/stromal tissue composition. Compared to deep endometriosis lesions, the endometrium displays larger and more uterine glandules during the secretory phase [58,59], increasing the expression of the senescence markers in epithelial cells and cytokines that are more closely associated with the secretory menstrual cycle phase than the proliferative phase [59]. Conversely, our results indicate that p16^Ink4a^ and lamin b1 are also expressed in non-epithelial cells, although at lower levels than in epithelial cells, suggesting that both epithelial and non-epithelial cells may play a role in senescence progression. Therefore, we hypothesize that in lesions that do not present glandular epithelial cells, the stromal cells display senescence characteristics, maintain endometriosis inflammatory milieu, and sustain lesion development.

As reviewed previously, several studies have demonstrated the difference between peritoneal fluid, follicular fluid, and serum interleukins in endometriosis and non-endometriosis groups [32]. However, the evaluation of tissue interleukin expression is still limited [6,60]. We observed higher IL-1β and IL-4 levels in endometriotic lesions than in eutopic endometria. IL-1β is a pro-inflammatory cytokine that mediates inflammatory processes and cellular proliferation. It is mostly produced by macrophages, monocytes, and fibroblasts induced by TNF-α, INF- α, INF- β, and INF- γ and acts in the early infection response. It is also a precursor of the senescent state. Although inconclusive results regarding IL-1β’s role in endometriosis have been published [61,62], increased IL-1 levels have been associated with major endometriosis-associated symptoms [32]. Few studies have linked IL-1β secretion with the induction of IL-17-producing cells [62,63], and thus the relationship between IL-1β and endometriosis may be related to IL-17 production.

A limitation of this study is the small sample size. In addition, despite having generated interesting primary results, these should be considered as preliminary and subject to confirmation. The non-endometriosis group consisted of patients with myoma and undiagnosed pain, which led us to analyze the results under the consideration that the differences encountered were between patients with possibly varying pelvic and inflammatory conditions; thus, the lack of differences between endometriosis and control groups, especially for peritoneal fluid evaluations, could be underestimated. To avoid subjective visual assessment and susceptibility to bias in the immunohistochemistry evaluation, we based our analysis on previous studies; however, we acknowledge that human visual interpretation and decision making were based on subjective criteria that can be difficult to reproduce [64].

Endometriosis is a disorder characterized by chronic inflammation, a feature also present in senescent cells. Although inflammation can help tissue regeneration and immune cell recruitment, the continuous presence of inflammation may be more hazardous than beneficial by promoting angiogenesis, DNA damage, and oxidative stress. By comprehending secretory phenotype in endometriosis tissues, it has become evident that deep endometriotic lesions have intrinsic controlling mechanisms. Such mechanisms may favor the formation of distinct microenvironments and the maintenance of the disease. The present study explores the hypothesis of a higher expression of senescence markers and SASP in endometriosis. Findings from the present study, for instance, lower lamin b1 and higher p16^Ink4^ and IL-1β in deep endometriotic endometriosis, and a pro-inflammatory milieu in the lesions and eutopic endometrium, mediated by the SASP, suggest that a portion of deep endometriotic endometriosis cells might be entering the senescence state, which may explain some key characteristics of the disease. The presence of senescence markers in lesions in both cell types (glandular and non-glandular), as well as in the eutopic endometrium, might help to evaluate if senescence features are present in endometriosis and if this characteristic would favor endometriosis maintenance. Additionally, this knowledge may pave the way to propose routes of delivery or new pharmaceutical approaches to control endometriosis symptoms and progression. Anti-aging molecules such as resveratrol, rapamycin, metformin, and aspirin could be investigated as potential therapeutics to target senescence pathways and alleviate symptoms. Further studies are necessary to confirm the accumulation of senescence cells in deep endometriotic endometriosis.

## 4. Materials and Methods

### 4.1. Patients and Ethics

This study is part of the Women’s Health Program of the Hospital Israelita Albert Einstein. Patients were selected between September 2017 and September 2019, screened by a single gynecologist surgeon (SP), and divided into two groups: with and without endometriosis. The study was approved by the Research Ethics Committee of the Hospital Israelita Albert Einstein (CAAE: 56229916.9.0000.0071). Written informed consent was obtained from all the patients.

The endometriosis group consisted of 27 patients with a proven histological diagnosis of endometriosis and 19 patients without endometriosis (non-endometriosis endometrium) (absence of endometriosis foci confirmed at the time of the surgical procedure). Our sample size was calculated with 90% power and an alpha-type error rate of 0.05. In these procedures, patients undergoing myomectomy surgery and pelvic pain diagnosis were included in the control group.

The inclusion criteria for both groups were as follows: age between 18 and 50 years; eumenorrheic menstrual cycles with the interval between cycles varying from 24 to 35 days [65]; and absence of hormonal therapy, including gonadotropin-releasing hormone analogs, progestins, and oral hormonal contraceptives, for six months before surgery. Patients who met one or more of the following criteria in the last three months before laparoscopy were excluded from the study: status as a smoker; presence of hydrosalpinx and endometrial polyps; diagnosis of diabetes mellitus or other endocrinopathies, cardiovascular diseases, dyslipidemia, systemic lupus erythematosus or other rheumatological diseases, or oncological diseases.

### 4.2. Study Design

To acquire a homogeneous group, the present study only selected deep endometriosis lesions. Endometriosis and non-endometriosis patients underwent video laparoscopy for resection of endometriosis lesions and myomectomy (intramural fibroids only) and investigation of pelvic pain. Deep retrocervical samples of endometriosis lesions were collected for this study. For both groups, the endometrium was collected shortly after the patient was positioned for the surgical procedure using a Pipelle curette. All collected tissues were stored at −80 °C, and immunohistochemistry biopsies were frozen in Tissue Tek medium. Peritoneal fluid samples were collected after visualization of the cul de sac at the beginning of the surgery to avoid blood contamination and divided into endometriosis peritoneal fluid and non-endometriosis peritoneal fluid. The biopsies collected from the endometriosis and non-endometriosis groups were divided into three groups: non-endometriosis endometrium (hereafter called the non-endometriosis group), eutopic endometrium (hereafter called the eutopic group), and endometriosis lesion (hereafter called the lesion group).

Senescence biomarkers (p16^Ink4a^, lamin b1, and IL-1β) were identified by immunohistochemistry, and the SASP was evaluated by Luminex MagPix immunological assay. The evaluated tissues included endometriosis lesions, eutopic endometrium and peritoneal fluid, and non-endometriosis endometrium and peritoneal fluid.

The proliferative and secretory phases were divided according to the beginning of the menstrual cycle provided by the patient. The proliferative phase was considered 0 to 15 days after the first day of the last menstruation, and secretory phase was considered 16 to 35 days after the first day of the last menstruation.

### 4.3. Immunohistochemistry

Immunohistochemistry was performed following the protocol from Dunstan et al. (2011) [66]. Tissue samples (4 μm thick) were prepared and mounted on slides. Staining was performed using a polyclonal antibody against lamin b1 (H90, catalog no. Sc-20682; Santa Cruz Biotechnology, Dallas, TX, USA) at a dilution of 1:100 and anti-p16 antibody (CINtec p16 Histology Ventana Roche—805-4713, Rotkreuz, Switzerland) at a concentration of 1.0 μg/mL. IL-1β was localized using a polyclonal antibody against IL-1β (H90, catalog no. Sc-20682; Santa Cruz Biotechnology, Dallas, TX, USA) at a dilution of 1:100. Photomicrography was performed with an IX-51 microscope (Zeiss, Oberkochen Germany).

Immunohistochemistry was performed using a BenchMark ULTRA IHC/ISH Marking Platform (Ventana Medical Systems, Oro Valley, AZ, USA). The UltraView Universal DAB Detection Kit (Ventana Medical Systems, Oro Valley, AZ, USA) was used to detect the marking signal. At the end of each stage, the platform underwent successive washes with Tris-based buffer solution (reaction buffer pH 7.6). An ultra-coverslip protection solution was used for permanent preservation.

For quantitative analysis, we used the histometric method, a semiquantitative subjective scoring system to evaluate the protein-positive areas. Four random areas from four different slides of the same tissue (a total of sixteen images per tissue) were analyzed. Our system was based on a previously reported manual scoring method [67,68]. The analysis of positive staining was validated by the method from Wang et al. (1998) [69], indicating a high correlation between the results obtained via objective computerized image analysis and subjective semiquantitative manual visualization. For histometric analysis, the IX51 inverted microscope was used to visualize, photograph, and quantify images with the Cell Sens Dimension software (Olympus, Center Valley, PA, USA). This program allowed us to select the areas to be quantified (area calculated in µm^2^). We indicated tissue areas (stained protein and cell cytoplasm) and non-tissue areas (areas on the slide without tissue).

### 4.4. Immunofluorescence

Endometriosis lesion, non-endometriosis, and eutopic endometrial tissue samples of 4 µm thickness were prepared and mounted on slides following an immunofluorescence protocol for co-localization of p16^Ink4a^ and lamin b1 with e-cadherin (epithelial cell marker). Briefly, samples were thawed, fixed in 4% paraformaldehyde, washed in PBS with 0.5% Tween, and immersed in Triton X-100 for better antibody perfusion. Subsequently, samples were incubated overnight with a monoclonal antibody against p16^Ink4a^ (1:100, rabbit monoclonal (EPR1473) to CDKN2A/p16^INK4a^ tag; Abcam, Cambridge, UK) or a polyclonal antibody against lamin b1 (1:100, H90, catalog no. Sc-20682; Santa Cruz Biotechnology, Dallas, TX, USA) and mixed with pre-diluted e-cadherin (Ref. GA059—Monoclonal mouse anti-human e-cadherin clone NCH-38 ready-to-use (Dako Omnis), Santa Clara, CA, USA). The slides were then washed in PBS with 0.5% Tween, and both secondary antibodies, Alexa Fluor^®®^ 488 (1:400, Goat Anti-Rabbit IgG H&L (ab150077) Abcam, Cambridge, UK) and Alexa Fluor^®®^ 594 (1:400, Goat Anti-Mouse IgG H&L pre-adsorbed (ab150116) Abcam, Cambridge, UK), were added. A qualitative analysis was performed using a Carl Zeiss LSM 710 confocal microscope (Oberkochen, Germany) fitted with Zen (2010) software (Zeiss, Oberkochen, Germany).

### 4.5. SASP

Endometriosis lesions, endometrium (eutopic and non-endometriosis), and peritoneal fluid were evaluated for cytokine expression using a multiplex immunological assay. The assay uses a magnetic bead system, the HCYTOMAG-60K MILLIPLEX MAP Human Cytokine/Chemokine Magnetic Bead Panel—Immunology Multiplex Assay kit (Sigma-Aldrich, Inc. St. Louis, MO, USA). The readings were obtained using a Millipex Map device (Merck Millipore^®®^, Burlington, MA, EUA). This panel was composed of the following cytokines, chemokines, and growth factors: epidermal growth factor (EGF), IL-1α, IL-1β, IL-2, IL-4, IL-6, IL-8, IL-10, IL-15, and IL-17A.

### 4.6. Statistical Analysis

Patient characteristics were described as absolute frequencies and percentages. Shapiro–Wilk tests were used to assess the distribution of all quantitative variables. Generalized linear mixed models with gamma or normal distribution were adjusted, considering the variables: groups (non-endometriosis, eutopic, and lesion) and menstrual cycle phase (secretory and proliferative). Results were presented as estimated means and 95% confidence intervals (CI) or mean ratios (MR) between groups and 95% CI for comparisons of interest. These models considered the dependence between measurements obtained from the same individual and within the same group, since we considered 16 replicates for each protein evaluation. For cytokines, the model analyzed the dependence between the measurements taken within the same group and, more broadly, within the non-endometriosis and endometriosis groups (eutopic and lesion) [70]. The level of significance adopted was 5%. R statistical package (R Core Team 2017) was used.

## Figures and Tables

**Table 1:** Correlation between age and BMI with protein expression in tissue samples.

Protein Expression (%)	MR (95% CI)	*p*-Value
IL-1β		
Age (years)	0.978 (0.925–1.034)	0.436
BMI (kg/m^2^)	1.015 (0.958–1.075)	0.618
		
p16^Ink4a^		
Age (years)	0.969 (0.921–1.019)	0.223
BMI (kg/m^2^)	1.020 (0.964–1.078)	0.493
		
Lamin b1		
Age (years)	1.003 (0.965–1.042)	0.893
BMI (kg/m^2^)	0.994 (0.937–1.055)	0.841

BMI, body mass index; MR, mean ratio; 95% CI, 95% confidence interval.

**Table 2:** Protein expression (p16^Ink4a^, lamin b1, and IL-1β) according to tissue type (control endometrium, eutopic endometrium, and endometriosis lesion).

Secretory Phase	MR (95% CI)	*p*-Value
IL-1β		
Non-endometriosis endometrium	0.890 (0.669–1.185)	0.426
Lesion	1.600 (1.206–2.122)	0.001 *
p16^Ink4a^		
Non-endometriosis endometrium	1.158 (0.885–1.515)	0.284
Lesion	1.567 (1.206–2.036)	0.001 *
Lamin b1		
Non-endometriosis endometrium	1.138 (0.898–1.442)	0.286
Lesion	0.816 (0.648–1.028)	0.084
Proliferative Phase	MR (95% CI)	*p*-Value
IL-1β		
Non-endometriosis endometrium	1.042 (0.798–1.360)	0.763
Lesion	1.720 (1.358–2.179)	<0.001 ^#^
p16^Ink4a^		
Non-endometriosis endometrium	0.649 (0.496–0.849)	0.002 ^#^
Lesion	1.182 (0.940–1.487)	0.154
Lamin b1		
Non-endometriosis endometrium	0.645 (0.508–0.820)	<0.001 ^#^
Lesion	0.643 (0.523–0.791)	<0.001 ^#^

MR, mean ratio; 95% CI, 95% confidence interval. Reference group was eutopic endometrium from endometriosis patients in the secretory and proliferative phase. * *p* < 0.05, compared to the eutopic endometrial group in the secretory phase. # *p* < 0.05, compared to the eutopic endometrial group in the proliferative phase.

**Table 3:** Correlation between protein expression and cytokine expression (pg/mL) according to tissue type (non-endometriosis endometrium, eutopic endometrium, and endometriosis lesion).

Secretory Phase	MR (95% CI)	*p*-Value
p16^Ink4a^		
IL-17A	1.036 (1.003–1.069)	0.034 *
Lamin b1		
IL-1A	0.999 (0.998–1.000)	0.039 *
IL-2	0.300 (0.101–0.893)	0.035 *

MR, mean ratio; 95% CI, 95% confidence interval. * *p* < 0.05 when compared to the eutopic endometrial group in the secretory phase.

## Data Availability

The data that support the findings of this study are available on reasonable request from the corresponding author. The data are not publicly available as they contain information that could compromise the privacy of the research participants.
